# The prioritization of symptom beliefs over illness beliefs: The development and validation of the Pain Perception Questionnaire for Young People

**DOI:** 10.1111/bjhp.12275

**Published:** 2017-10-09

**Authors:** Daniela Ghio, Wendy Thomson, Rachel Calam, Fiona Ulph, Eileen M. Baildam, Kimme Hyrich, Lis Cordingley

**Affiliations:** ^1^ Division of Musculoskeletal and Dermatological Research School of Biological Sciences Faculty of Biology, Medicine and Health Manchester Academic Health Science Centre University of Manchester UK; ^2^ NIHR Manchester Biomedical Research Centre Central Manchester University Hospitals NHS Foundation Trust Manchester Academic Health Science Centre University of Manchester UK; ^3^ Arthritis Research UK Centre for Genetics and Genomics Centre for Musculoskeletal Research Manchester Academic Health Science Centre University of Manchester UK; ^4^ Division of Psychology and Mental Health Faculty of Biology, Medicine and Health Manchester Academic Health Science Centre University of Manchester UK; ^5^ Alder Hey Children's NHS Foundation Trust Liverpool UK; ^6^ Arthritis Research UK Centre for Epidemiology Centre for Musculoskeletal Research Manchester Academic Health Science Centre University of Manchester UK

**Keywords:** adolescence, illness representations, pain cognitions, self‐regulatory model

## Abstract

**Objectives:**

To investigate the suitability of the revised Illness Perception Questionnaire (IPQ‐R) for use with adolescents with a long‐term pain condition and to validate a new questionnaire for use with this age group.

**Design:**

A three‐phase mixed‐methods study.

**Methods:**

Phase 1 comprised in‐depth qualitative analyses of audio‐recorded cognitive interviews with 20 adolescents with juvenile idiopathic arthritis who were answering IPQ‐R items. Transcripts were coded using framework analysis. A content analysis of their intended responses to individual items was also conducted. In Phase 2, a new questionnaire was developed and its linguistic and face validity were assessed with 18 adolescents without long‐term conditions. In Phase 3, the construct validity of the new questionnaire was assessed with 240 adolescents with juvenile idiopathic arthritis. A subset of 43 adolescents completed the questionnaire a second time to assess test–retest reliability. All participants were aged 11–16 years.

**Results:**

Participants described both conceptual and response format difficulties when answering IPQ‐R items. In response, the Pain Perception Questionnaire for Young People (PPQ‐YP) was designed which incorporated significant modifications to both wording and response formats when compared with the IPQ‐R. A principal component analysis of the PPQ‐YP identified ten constructs in the new questionnaire. Emotional representations were separated into two constructs, responsive and anticipatory emotions. The PPQ‐YP showed high test–retest reliability.

**Conclusions:**

Symptom beliefs appear to be more salient to adolescents with a long‐term pain condition than beliefs about the illness as a whole. A new questionnaire to assess pain beliefs of adolescents was designed. Further validation work may be needed to assess its suitability for use with other pain conditions.

**Statement of contribution**

***What is already known on this subject?***

- Versions of the adult Revised Illness Perception Questionnaire (IPQ‐R) have been adapted for adolescents and children by changing item wording; however, research to assess the degree to which the underlying IPQ‐R constructs are relevant to adolescents with a long‐term condition had not been performed.

***What the present study adds?***

- In adolescents, beliefs about symptoms of their condition are more salient than beliefs about the illness as a whole.
- Question response formats for children and young people need to take account of age‐specific abilities.
- A new questionnaire has been designed for adolescents with pain. It is theoretically congruent with the CS‐SRM.

## Background

Illness perceptions play an important role in determining health outcomes in those with long‐term conditions (Hagger & Orbell, 2003). The Common Sense Self‐Regulatory Model (CS‐SRM) proposes that individuals create ‘mental representations’ of a perceived health threat and that these shape their behavioural and emotional responses to a threat such as a diagnosis of illness (Leventhal, Leventhal, & Contrada, 1998). In adults, mental representations have been assessed across a wide range of conditions with different versions of the Illness Perception Questionnaire (IPQ); the original IPQ (Weinman, Petrie, Moss‐morris, & Horne, 1996), the Revised Illness Perception Questionnaire (IPQ‐R; Moss‐Morris *et al*., 2002) and the Brief IPQ (Broadbent, Petrie, Main, & Weinman, 2006).

To date, there has been little research exploring the extent to which health psychology theories developed with adults such as the CS‐SRM can be successfully operationalized and applied to children or adolescents (Bogosian, Van Vliet, Craig, Fraser, & Turner‐Cobb, 2016; Law, Tolgyesi, & Howard, 2014). A small number of studies have been conducted into the illness representations of children and young people with a variety of long‐term conditions including asthma (Walker, Papadopoulos, Lipton, & Hussein, 2006), diabetes (Skinner *et al*., 2003), and cystic fibrosis (Bucks *et al*., 2009); however, it is noticeable that in most of these studies, the adaptations to the measures of illness representations were linguistic or condition specific rather than conceptual (Law *et al*., 2014).

Juvenile idiopathic arthritis (JIA) is the most common inflammatory arthritis in children and young people. It is characterized by relapsing–remitting episodes of disease activity in which joints can become swollen and limited in movement. Pain may be experienced almost daily (Schanberg, Anthony, Gil & Maurin, 2003) and is not fully explained by disease activity alone (Rapoff & Lindsley, 2011). For some individuals, pain continues even during periods when underlying disease activity is low (Lomholt, Thastum, & Herlin, 2013; Thastum & Herlin, 2011). Researchers investigating the beliefs of adolescents with chronic pain conditions have largely used the fear‐avoidance model and focused on pain catastrophizing, a maladaptive cognitive process in which the perceived danger associated with pain is magnified, and generates a fear response increasing the likelihood of avoidant and hypervigilant behaviours (Vlaeyen & Linton, 2000). The fear‐avoidance model (Vlaeyen & Linton, 2000) emphasizes that the problematic responses to pain stimuli occur as a result of *overestimating the degree of danger*. In contrast, the authors of CS‐SRM asserted that fear alone does not lead to the instigation of new behavioural responses. Instead, they argued that behavioural and emotional responses are dependent on how the health threat is conceptualized across a range of domains, not just with danger (Leventhal, Bodner‐Deren, Breland, Hash‐Converse, & Phillips, 2012). The roles of other pain beliefs held by adolescents such as pain controllability, cause, or chronicity have not been widely studied.

A recent longitudinal study comparing two models (the fear‐avoidance model with the CS‐SRM) for their ability to predict disability in adult patients undergoing an intervention for back pain found that adaptive illness perceptions were stronger predictors of positive change in disability (Bishop *et al*., 2015). A key finding from that study was that while these psychological theories may overlap, the pain cognitions associated with the CS‐SRM were stronger predictors of outcome.

Despite the potential of the CS‐SRM framework for understanding, modelling, and predicting responses to a long‐term condition especially pain conditions, testing of the underpinning constructs for relevance to adolescents has not been conducted. This is an important omission because adolescents may conceptualize their illnesses differently to adults, either due to differences in cognitive developmental processes or due to the different roles they have in self‐management. Law *et al*. (2014) undertook a review of the literature into illness beliefs of children and young people and found that relationships between beliefs relating to personal control and self‐management, which had been identified in adults, were not replicated in younger people. The review's authors suggested that the findings could be attributable to the social context of young people's illnesses, especially the role parents' play. The aims of this study were, therefore, to investigate the extent to which adolescents' illness beliefs could be assessed using the IPQ‐R and to then develop a valid and reliable theory‐based measure of adolescents' illness representations of their long‐term condition.

## Methods

### Design

There were three phases in this study comprising a preliminary qualitative phase followed by two validation phases. The aim of Phase 1 was to assess the degree to which the belief domains of the (adult version) Revised IPQ were relevant to adolescents with a long‐term condition (in this case Juvenile Idiopathic Arthritis, or JIA) and to assess the response options for suitability for completion by adolescents. To do so, the first phase included a cognitive interviewing study by undertaking one‐to‐one interviews using both framework and content analysis. The Revised IPQ rather than the Brief version BIPQ (Broadbent *et al*., 2006) was chosen as it includes a range of items to assess each construct which facilitated exploration of the constructs during cognitive interviewing. The aim of the second phase was to devise the new questionnaire and assess its face validity. To address this second phase of development, the preliminary draft of the PPQ‐YP was sent to adolescents without JIA to answer the questionnaire and provide feedback on the language and length. The third phase was the main validation study of the new questionnaire following modifications resulting from data acquired in earlier phases. This was a survey‐based questionnaire validation. This study used a postal survey to recruit a clinical JIA population who completed the questionnaire. A subset of 76 comprised the first wave of posting, and this group was invited to complete a second time after 2 weeks to assess the retest reliability. A further 161 completed the questionnaire in the second wave of recruitment.

### Participants

Three different samples of participants were involved in each of the three phases. Participants previously recruited into a national prospective study of outcomes for children and adolescents with JIA, the Childhood Arthritis Prospective Study (or CAPS; see [Hyrich *et al*., 2010]), were recruited for Phase 1. Eligibility for participation in Phase 1 was limited to those aged 11–16 attending one of the hospitals located close to the study base and with routine appointments during a specified 4‐month period. The criteria for referring patients to that hospital were the same as those attending all the other tertiary centres involved in the wider CAPS study. The centre used for recruitment was similar in terms of size of other rheumatology clinics. Of 60 eligible participants, 25 were initially recruited; however, four subsequently cancelled their clinic appointment and one participant could not complete the interview due to time constraints. This resulted in a final sample of 20.

Participants for the Phase 2 face validity study were an opportunity sample of adolescents without JIA aged 11 and 16 years. Twenty healthy adolescents without chronic pain were recruited by advertisement with 18 participants providing full data.

Participants in the Phase 3 validation study were recruited from eligible CAPS study participants in two waves. All had a diagnosis of JIA and were aged between 11 and 16 years at the time of recruitment. In the first wave, two hundred and twenty‐one (221) adolescents who had a scheduled annual study appointment between October 2013 and January 2015 were invited to complete the questionnaire. Seventy‐nine responded, a response rate of 38%. Of these 79 adolescents, 72 consented to repeat the questionnaire 2 weeks later. Forty‐three adolescents returned the repeat questionnaire. In the second recruitment wave, from January 2015 to January 2017, an additional 407 adolescents were invited to complete the new questionnaire with 161 adolescents completing the questionnaire as part of a large questionnaire packs. This means a total of 240 adolescents completed the new questionnaire at one time point.

The study was conducted in accordance with the Declaration of Helsinki and was approved by an NHS Research Ethics Committee. Prior to participation in each study, participants aged 16 and above gave informed consent and those aged under 16 gave informed assent with parents/guardians providing informed consent.

#### Phase 1: Cognitive interviewing procedure

Cognitive interviewing is a method used to elicit and assess the cognitive processes individuals are engaged in when they are answering a questionnaire and reveal the mental constructs they are drawing on in order to formulate their responses (Willis, 2004; Willis, Royston, & Bercini, 1991). These include the degree to which the individual comprehends items (overall item intent as well as words meaning), the decision‐making processes involved in providing a response (taking into account motivation and social desirability), and the degree to which their own responses can map onto the response options available. Two cognitive interviewing techniques were used, ‘think‐aloud’ and ‘verbal probing’ (Willis, 2004). Asking a participant to ‘think‐aloud’ or verbalize their thoughts as they answer an item enables the recording of some cognitive processes. Verbal probing involves the interviewer asking for specific information relevant to the item either concurrently or at the end of the interview.

Interviews took place before or after participants' routine clinic appointments in a separate clinic room. To build trust and rapport, the interviewer was introduced to the participants by the research nurses who were all known to the participants. In the majority of cases, the parents/guardians of the participants remained in the clinic room during the interviews however they did not participate in any way as they were occupied with a task for an unrelated study.

All participant documentation (information sheets, consent forms, questionnaires and written interview notes) were given a unique identifiable number. During transcription, all personal information and identifiable characteristics were deleted, and after analysis, participants' names were replaced with pseudonyms.

The cognitive interviews began with the opportunity to practice the ‘think‐aloud*’* procedure with a neutral task prior to the main interview. The cognitive interviews included a combination of think‐aloud and verbal probing techniques used by the interviewer while participants responded verbally or in writing to items from the IPQ‐R. Some IPQ‐R items had been modified slightly to make them suitable for adolescents as was performed in previous studies using the IPQ (e.g., Walker *et al*., 2006). However, this was the first study to test the construct validity of an illness perceptions questionnaire as well as the theoretical framework with this population. A probing protocol was developed in advance with additional spontaneous probes used by the interviewer to encourage the participant to ‘think‐aloud’ about their responses or for clarification. After the interviews, participants were given the opportunity to ask questions or to add further ideas.

Analysis of the data set involved two different techniques. Framework analysis (Ritchie & Lewis, 2003) was used to assess the degree to which the domains of the IPQ‐R mapped onto the constructs used by adolescents. Both ‘top‐down’ and ‘bottom‐up’ approaches to data interpretation were undertaken. Top‐down refers to the use of the a priori theory (in this case CM‐SRM). The bottom‐up analysis enabled the inclusion of items which did not fit CS‐SRM. This approach was sufficiently flexible to include novel themes to be identified. Management of the interview data consisted of charting data according to concepts and domains outlined by the CS‐SRM using the software NVivo 10 (QSR International, 2012).

The transcripts were indexed using a combination of CS‐SRM domains (identity, timeline, consequences, coherence, cause, emotional representations) and other themes arising from the interviews. Data were charted, mapped, and interpreted. During these activities, patterns or links in the data identified interpreted to create major themes and subthemes. Interpretations and coding were discussed by the team to strengthen dependability, confirmability and credibility of the data. An iterative process in reorganizing the data within the parent themes led to a coherent description of each construct. Two of the study team continued to return to the transcripts with the thematic framework after discussions to ensure that it reflects the data. The narrative account allowed a comparison of the constructs with the literature supporting the CS‐SRM domains and led to the development of a framework which reflected the responses of this adolescent sample.

Latent content analysis (Krippendorff, 2012) was undertaken to investigate the suitability of the IPQ‐R response options when endorsing specific responses to IPQ‐R items. For the latter, a coding manual was developed using three participants' transcripts selected at random. Codes were generated from issues participants identified as problematic when responding to IPQ‐R items. The codes were discussed and revised by two authors and then coded independently. The level of agreement between the raters measured by inter‐rater reliability was found to be high (κ = .86, *p *<* *.001) (McHugh, 2012). The few discrepancies that occurred were reviewed by remaining authors. The final coding manual included five codes presented in Table 1.

**Table 1:** Code manual developed for the content analysis

Code	Definition
Not relevant	The participant claimed the item was not relevant to them
Confusion	The participant was confused, asked to repeat the question or had to skip it because they did not understand the item
Incongruent endorsement	The written endorsement differed from their verbal response to the item
Incongruent answer	The verbal response did not answer the question in the item
No difficulty	The participant had no difficulties responding to the item

Findings from these two sets of analyses of the cognitive interviews were then used to develop items for a pilot version of the questionnaire which was named the Pain Perception Questionnaire for Young People or PPQ‐YP. The development of the PPQ‐YP addressed two areas: The first was the selection and wording of the items and the second was the response formats and scoring. There were originally 61 items separated into four sections. Sections were created to facilitate responses with different sections addressing different constructs, styles of question, and/or response formats. The response formats and the items were tested in the next phase. The cause subscale of the PPQ‐YP combined aspects of the full adult version of the IPQ with those of the Brief IPQ in terms of response format in that it allows the participants to identify three causal beliefs (Broadbent *et al*., 2006).

#### Phase 2: Face and linguistic validity procedure

The sample of healthy adolescents completed the PPQ‐YP. As they did not usually experience pain, they were asked to use a recent occurrence of pain in order to respond to items. This enabled us to check that the language of the PPQ‐YP was accessible to those who were not used to having their pain levels assessed, in contrast to participants in Phase 1 who were all diagnosed with JIA. The sample of healthy adolescents also provided written responses to questions about clarity of the PPQ‐YP, for example, if there were any words or terms that were difficult, and if there were questions they did not understand. They also had the opportunity to add any other comments. These data were analysed and relevant revisions were made to items in a revised version of the PPQ‐YP, and this subsequent version was utilized in the main validation study.

#### Phase 3: Preliminary validation procedure

Eligible patients were sent the revised version of the PPQ‐YP (See Data S1) plus a pain visual analogue scale (Pain VAS; 0–100 mm). Those consenting to be contacted for the follow‐up phase were sent the same questionnaire 2 weeks later. There were no significant differences in sex, physical functioning (scores from the Childhood Health Assessment Questionnaire), or mood (scores from the Mood and Feelings Questionnaire) between those participants taking part in the study who completed the questionnaire and eligible participants who were sent the questionnaire but did not complete it (see Table 2). However, the group that did not complete the PPQ‐YP was slightly older than the group that did.

**Table 2:** Demographic characteristics for participants in Phase 3. Comparison of the completers and non‐completers of the PPQ‐YP in Phase 3

Demographic characteristics	Completed PPQ‐YP (*N* = 256)	Not completed PPQ‐YP (*N* = 372)	Mann–Whitney *U* test (*p*‐value)
Female *N* (%)	171 (67%)	234 (63%)	.36
Age mean (*SD*)	15 (3.1)	15.9 (3.3)	<**.001**
Childhood Health Assessment Questionnaire (CHAQ) Mean (*SD*)	0.85 (0.8)	0.9 (0.7)	.1
Mood and Feelings Questionnaire (MFQ) Mean (*SD*)	14 (11.9)	15 (11)	.4

*Note*. Bold values are significant.

To identify a factor structure the first‐time, PPQ‐YP responses were used in a principal component analysis (PCA) using oblique rotation (direct oblimin) which allows factors to be correlated. Test–retest reliability was assessed by sending 72 participants a second copy of the questionnaire 2 weeks later, of whom 43 completed it. Those who completed the questionnaire a second time were slightly older than those who did not (mean age 14.28 years [*SD* = 1.35] compared to 13.93 years [*SD* = 1.56], respectively). The proportion of females responding was slightly less than the non‐responders (63% compared with 69.7%). Related Wilcoxon signed‐ranks test (Wilcoxon, 1946) was used to assess the difference between scores over the 2‐week period. The interclass correlations (ICC) were used to test the reliability of the subscales of the PPQ‐YP completed at the two time points. Pain VAS scores were used to identify whether participants' pain levels had changed between PPQ‐YP administrations in case there were significant changes in the PPQ‐YP scores. To also test the stability of the PPQ‐YP over this period, Spearman's Rho was calculated to test the correlations in each subscale over the retest period. To test the validity of the identity subscale, the same approach to validation was undertaken as that used by Moss‐Morris *et al*. (2002) when developing the Revised IPQ. A paired‐samples *t*‐test was conducted to test the difference between the symptoms experienced and whether they were identified by the respondents as being associated with pain. This analysis was repeated with symptoms associated with treatment.

## Results

### Phase 1 findings: Framework analysis

The framework analysis helped to establishing whether the adolescents' beliefs about pain could be mapped to the original constructs proposed by the theoretical framework of the CS‐SRM. The analysis led to additions or changes to items to reflect the adolescents' conceptualization of their condition identified in the analysis. The major change was the shift from assessing illness beliefs to pain beliefs. This occurred because during the interviews, it became very apparent that the adolescents' responses were principally driven by their pain perceptions rather than their views and reactions to having JIA.

The analyses below are presented in an order which reflects the proposed changes to the questionnaire.

#### Adding items to include different environments

For the domain of *consequences,* the adolescents indicated that pain impacts on different aspects of their lives. For example, the adolescents believed that pain impacted them differently in school compared to at home, and how they coped with pain was dependent on the environment. For adolescents like Kevin (aged 11), the impact in school is higher than at home ‘I can't like run or play football or do stuff sometimes I could walk and write and all that’. This led to adding items specifying different environments that pain may impact, including school, relationships, home, and family life under the construct of *consequences*.

#### Changed responses to timeline items

The construct of *timeline* refers to beliefs about the likely duration or temporal pattern of pain. In some participants, these beliefs appeared to be dependent on their perceptions of improvements in their pain. For example, Fay's (aged 11) beliefs about a short duration had changed following a specific experience; ‘When they took me off all my medicine erm they tried me off it and it [the pain] came back so I am not sure, kind of sure’. The adolescents did not recognize a temporal pattern such as relapsing–remitting cycle of pain. Instead, they viewed pain as ‘unpredictable’. However, they did describe adapting normal behaviours such as preparing for a ‘pain day’. *F*or some adolescents, this meant adapting to all or nothing behaviour patterns in which they would ‘overdo’ activities but anticipate and plan high pain the next day. This led us to develop new responses to the *timeline* items of the questionnaire and new items to capture the beliefs expressed.

#### Adding items to distinguish between personal and treatment control

The adolescents' beliefs about personal and treatment control were not captured using the existing items. For example, the control that treatment afforded over their pain was limited. Carrie aged 12, said that ‘I have bad days with my treatment and then I have good days where the good days are I can't feel the pain and I can get on with my sports and my life and that. Some days I can feel the pain and I have to sit down and be steady with myself and lie down’. However, the key was that the adolescents recognized feeling personal control over when and how they accessed treatment. New items were therefore added to distinguish beliefs about personal control over pain, personal control over treatment, and how treatment can control pain.

#### Adding items to identify gaps in coherence

The adolescents reported feelings of uncertainty about pain and identified many limitations in their understanding of pain and why it occurs. Their uncertainty appeared to be an additional source of concern. The existing IPQ‐R illness coherence domain includes items to assess an individual's ability to hold a coherent representation of a health threat, that is, sets of beliefs which have an internal logic or consistency. However, the adolescents found the wording vague and the items did not reflect capture the adolescents' struggles to understand pain including the rationale for or mechanisms of treatment. For almost all the participants, pain was a daily occurrence and yet they believed they did not understand their pain or how it related to their underlying condition. For example, Daisy aged 13 said ‘I understand what it is and that it makes everything swell up and like hurt but some stuff I don't know like why it suddenly can just start hurting for no reason*’*. New items were therefore added to this section to assess illness coherence and understanding.

#### Adding treatment attributions underlying symptoms

The construct of *illness identity* assesses knowledge about the ‘label’ and the related symptoms that are attributed to the condition. For some adolescents, certain symptoms were not attributed to the condition but rather were related to treatment. Symptoms such as *lost or gain weight, unwell,* and *felt like vomiting* were all perceived as caused by side effects from the medication. The adolescents map causal attributions to the symptoms. Ian, aged 15, said that *feeling unwell* was ‘because of medication’. Similarly, Wyatt, aged 13, and Ian attributed *gaining or losing weight* to medication. This led to adding a second column to the *identity* section to assess symptom attributions to treatment which is similar to more recent versions of the adult IPQ‐R.

#### Changed causal attributions to recent pain experience

When being interviewed about symptoms, some participants viewed pain caused by ‘doing too much’. For example, Gwen, aged 11, said that she feels pain ‘because I've done too much*’* and later she attributed *feeling tired* to arthritis because ‘when walking, feet hurt because of arthritis*’*. For adolescents like Gwen, pain had a purpose. It was an indicator of the personal limits set by her condition. However, the cause of the pain was not arthritis per se, but rather a result of her behaviour, in this case ‘doing too much’. Furthermore, adolescents expressed beliefs that the cause of pain can change over time. For example, Ian, aged 15, who said ‘I don't think there is a logical information that stress or worry had caused it. I got [it] when I was younger and I was never stressed or worried when I was younger. I am not sure to be honest, it's just it could be now, it could be involved. Stress or worry could be involved in triggering it so when I get stressed a lot it's just, it could be painful’. Therefore, to capture these important views, the causal attributions section has been changed and now the item asks about the perceived causes of the most recent pain experience.

### Phase 1 findings: Content analysis

The wording and the response options of new PPQ‐YP items were further informed by the content analysis of the cognitive interview data. The finalized code manual consisted of five codes which are given in Table 1. (The details of the coding of each IPQ‐R item are given in Table  S1).

There were recurring difficulties with negatively worded questions such as ‘Nothing I do will have any effect on my arthritis’. The interviewer identified delays in replying and needed to repeat these items frequently. Some of the participants spontaneously identified this as a problem during the course of the interviews. Most of the problems under the ‘incongruent endorsement’ category in which the written response contradicted their verbal response occurred in relation to answers to negatively worded items.

They [items] are a bit confusing at times. ‘Cause you obviously have to think quite hard. Paige, (age: 15), (about the negatively worded items in personal control)

Cause when you asked me it [the item] I had to think if I say disagree does that mean that I didn't know anything about it or did it mean I did know something about it. Ian, (age: 15), (item ‘I don't understand my arthritis’)

The items associated with the next highest frequency of difficulties were those addressing beliefs about *consequences*. The existing items include examples of consequences which the adolescents may not have experienced, and they were unwilling to respond to what seemed like hypothetical questions with no experience on which to base their responses.

Erm I am not too sure about that one. Because I don't really know whether it does because I don't ask people about it so I don't know whether it does or does not. Eleanor (age: 14)

The items assessing the construct of *emotional representation* had the fewest problems in answering or understanding the questions regarding their emotional states. However, the adolescents' reasoning underlying their answers suggested that both the questions and the answers did not reflect their emotional representations of their pain. They struggled to choose a response that signified *how much* and *how often* they felt an emotion. For example, they justified choosing ‘disagree’ over ‘strongly disagree’ if it was an emotion they might have felt but do not feel it frequently.

I don't think about it when there is no pain I only think about it when there is pain. When I do have pain it just makes me sad makes me upset about it down about it I can't do anything for a while Ian (age: 15)

### Phase 2 results: Language validity – face validity study

The outcome of the analysis in Phase 1 was the development of the Pain Perception Questionnaire for Young People (PPQ‐YP). In the preliminary version of the PPQ‐YP, there were a number of words or concepts that were difficult for the participants. Table 3 summarizes those items that required further clarification. All participants reported completing the questionnaire within 10 min suggesting that its length was not a burden. Furthermore, none of the participants reported difficulties in understanding or using the revised response formats. A few minor changes were made to reflect the main comments (presented in the final column of Table 3), and this 61 item version of the PPQ‐YP was used in Phase 3.

**Table 3:** Changes for PPQ‐YP version 2 (Phase 2): Problems with items from the first version of the PPQ‐YP and changes made for the second version of the PPQ‐YP

Item	Issue	Outcome
Instructions – on the first page	Include ‘how you feel’ instead of just saying ‘your views’	We are interested in your views and how you feel about pain you may have relating to your arthritis
Tightness (symptom)	Not understood	‘Feelings of tightness in my body’
I believe having pain makes my family spend more money	Unclear	I believe my family spend more money because I have pain
Things I do now can affect my future pain	Unclear	There are things I do now which can affect whether I have pain in the future
Smoking/drinking causal attribute	Not relevant	Any other cause that you think of
Give an example to section D	Hard to follow instructions	Provided an example in section D to explain what to do in Section D

### Phase 3 results: Validation of the pain perceptions questionnaire for young people

#### Principal Components Analysis (PCA)

All of the 32 items related to Timeline, Consequences, Cure/Control, and Illness Coherence (see Table 4) were subjected to PCA with oblique rotation (Kaiser, 1974) using SPSS version 22. Complete‐case method was used to deal with missing values in items of all sections of the questionnaire as this method is the most conservative. The total complete‐case sample size was 196 participants. Prior to performing PCA, the suitability of data for factor analysis was assessed. This analysis had a Kaiser–Mayer–Olkin value of 0.887, still exceeding the recommended value of .6 (Kaiser, 1974) and Bartlett's Test of Sphericity (Bartlett, 1954) was statistically significant (*p* < .001), supporting the factorability of the correlation matrix. The PCA was run with a five components solution explaining a total of 55.9% of the variance.

**Table 4:** Principal component analysis of cognitive representations items

	I	II	III	IV	V
Component 1 α* = *.88					
**Consequences**					
I believe my pain causes problems for my family	0.77				
I believe having pain makes my family spend more money	0.75				
When I have pain, it affects how I am at home	0.79				
When I have pain, it affects me at school such as school work, school friends	0.80				
I believe my pain affects what other people think of me	0.70				
I believe having pain makes the hospital spend a lot of money	0.67				
When I have pain, it stops me from taking part in activities such as PE	0.72				
When I get pain, it makes me think my pain is *[endorsement is how serious amount]*	0.59				
When I get pain, I think my pain will improve	0.58				
Component 2 α* = *.88					
**Treatment and personal control**					
My treatment helps my pain get better		0.83			
I can continue with my activities because of my treatment		0.83			
Taking my treatment means I have *amount control*		0.77			
My treatment protects me from pain		0.76			
There are things I can do to make my pain better		0.63			
I am in control of my treatment for my pain		0.65			
This is the amount of control I feel I have over my pain *amount control*		0.54			
I can do a lot to control my pain		0.50			
Component 3 α = .80					
**Coherence**					
I understand my pain clearly			0.84		
When I have pain, I understand what causes my pain			0.78		
I understand how my treatment for pain works			0.67		
I do not have any questions about my pain			0.70		
I feel confused about why I get pain (reverse)			0.63		
Component 4 α = .76					
**Pain recurrence**					
My pain comes and goes				0.74	
My pain changes every day				0.65	
I believe I will keep having pain when I am an adult				0.63	
Over time I am getting pain more often				0.53	
When I get pain, it lasts a long time				0.50	
I believe I will stop getting pain soon				0.37	
Component 5 α *= *.65					
**Pain predictability**					
I can see a pattern in how and when I get pain					0.82
I can predict when I get pain					0.79
My behaviour can affect how much pain I have					0.47
There are things I do now which can affect future pain					0.48

The final 32 items loaded on five components that could be mapped to some of the original IPQ‐R construct domains. The first component was similar to the original IPQ‐R *consequences* domain*,* which included items originally designed to assess beliefs about the consequences of a health threat. The second component had item loadings that relating to personal and treatment control. For the PPQ‐YP, these items loaded strongly on the same component, suggesting that the items are assessing the same domain of *control*. The third component had all the items related to *pain coherence* loading strongly. The fourth component included items from what previously was identified as timeline*;* however, the items reflected the recurring nature of JIA, and therefore, this domain was renamed as *pain recurrence*. The fifth component included items assessing beliefs about predicting pain and identifying aspects affecting pain and therefore has been named *pain predictability*.

A separate PCA was undertaken with the items assessing emotional representations (see Table 5). Inspection of the correlation matrix revealed the presence of many coefficients of .5 and above. The Kaiser–Mayer–Olkin value was .89, exceeding the recommended value of 0.6 (Kaiser, 1974) and Bartlett's Test of Sphericity reached statistical significance (*p* < .001), supporting the factorability of the correlation matrix.

**Table 5:** Principal component analysis of emotional representations items

	I	II
Responsive α = .95		
Anger: How much	0.86	
How often	0.87	
Frustrated: How much	0.85	
How often	0.87	
Upset: How much	0.87	
How often	0.88	
Down: How much	0.89	
How often	0.87	
Anticipatory α = .95		
Afraid: How much		0.90
How often		0.87
Worried: How much		0.90
How Often		0.91
Anxious: How much		0.89
How often		0.87

Note

α is internal consistency of the subscales.

This analysis revealed the presence of two components. The first explained almost 65% of the variance while the second component explained 11% of the variance, the two components are strongly correlated (*r* = −.68). The emotions of anger, frustration, and feeling down or sad were grouped in the first component and labelled responsive emotions. Emotions relating to anxiety and fear were grouped together in the second component, and these were labelled anticipatory emotions.

##### Identity and cause subscales

The identity subscale contained two components, symptoms associated with illness and symptoms associated with treatment. Table 6 shows that all the symptoms were endorsed, supporting the decision to include this selection of symptoms in the PPQ‐YP. Interestingly, not all were associated with either pain or treatment. *Joints stiff* and *joints sore* as well as *tired* were the most frequently endorsed symptom with 65.0%, 62.9%, and 60.0%, respectively. *Cannot breathe well* was endorsed by 12.1% and was endorsed as associated with either pain or treatment by <3% of the participants.

**Table 6:** Frequencies in the Identity Subscale (Frequency of symptoms that were endorsed and the frequency of symptoms associated with pain and those symptoms associated with treatment, *n* = 240)

	Freq of symptom			Freq associated with pain			Freq associated with treatment		
	Yes	No	Missing	Yes	No	Missing	Yes	No	Missing
Vomiting	59	160	21	7	43	9	45	13	1
Cannot breathe well	29	192	19	6	25	0	3	24	2
Weight change	67	153	20	17	46	4	27	35	5
Tired	144	79	5	69	66	9	47	79	18
Joints stiff	156	65	19	139	17	0	13	102	41
Joints sore	151	73	16	142	9	0	14	91	46
Sore eyes	40	182	18	14	24	2	6	26	8
Feeling unwell	90	134	16	30	52	8	46	36	8
Headaches	83	141	16	23	49	11	21	50	12
Not sleep well	87	136	17	41	45	1	21	50	16
Upset tummy	59	161	20	13	40	6	30	29	0
Felt dizzy	42	180	18	8	30	4	15	24	3
Felt weak	70	153	17	44	19	7	24	33	13
Feel tightness	53	168	19	40	12	1	8	28	17
Change mood	94	127	19	50	36	8	35	46	13

The results of the paired‐samples t‐test showed a significant difference between the symptoms patients experience compared with the symptoms associated with pain, *t* (7.20), *p* < .001, and with treatment, *t* (7.96), *p* < .001. There was also a significant difference between symptoms associated with pain and those associated with treatment, *t* (3.73), *p* < .001. These results indicate that individuals held different views about symptoms of JIA, symptoms associated with pain, and those associated with treatment.

To validate this subscale, the frequency of a cause according to importance (endorsed 1, 2, or 3) and overall frequency of endorsement of cause regardless of rating were calculated. All of the causes were endorsed, most rated as a second or third cause. As shown in Table 7, the mostly frequently endorsed causes were stress or worry (38%), luck (33%), genetics (33%), immune system (32%), and doing too much (32%). That particular data set was incomplete with a large proportion of missing data (53%). This proportion of incomplete data may indicate problems with this subscale for adolescents. Psychological causes were the least likely to be endorsed and the least likely to be rated as the most important cause. The option *other* was endorsed (14%) with the open text indicating that *weather* was the main reason (21%), others included *strenuous activity* (8%), *swelling* (5%), *being taken off medication* (5%), other conditions such as *fracture,* and *hypermobility* (5%), *puberty* (2%)*, tiredness* (2%), and *arthritis* (2%).

**Table 7:** Frequency of causal beliefs

	Cause 1	Cause 2	Cause 3	Total
Stress or worry	56	18	18	92
Genetics	42	25	12	79
A germ or virus	16	20	15	51
Diet	2	9	11	22
Luck	25	25	29	79
Poor health	6	3	3	12
Pollution	–	2	2	4
Behaviour	2	6	6	14
Attitude	3	1	2	6
Family problems	3	5	1	9
Doing too much	27	24	25	76
Feeling down	–	2	6	8
Getting older	2	7	10	19
Accident	4	4	2	10
Type of person	–	1	2	3
Immune	27	28	21	76
Other	11	9	14	34
*Missing*	14	51	61	126

##### Internal consistency

Those items that are negatively related to other items were reverse‐scored prior to the calculation of the internal consistency (Field, 2013). The Cronbach alpha value represents the degree to which they measure the same underlying construct (Cronbach, 1951). Values of .7 to .8 are considered acceptable for psychological constructs (Field, 2013). Tables 4 and 5 show that subscales demonstrated good internal consistency with scores ranging from .65 to .95.

##### Test–retest reliability

To account for changes in pain, a pain VAS was included at both time points (see Table 7), and while the follow‐up scores were highly correlated with baseline scores (*r*_s_ = .69, *p *<* *.001), a Wilcoxon signed‐ranks test (Wilcoxon, 1946) indicated that follow‐up scores were not statistically higher than time 1 (*Z* = −1.10, *p *=* *.274). When the pain scores at 2‐week follow‐up were subtracted from the pain score at baseline, the median difference was 0 (IQR: −17.50 to 2), with the score differences ranging from −71 to 43 mm.

The test–retest reliability was assessed using two recommended methods (Streiner, Norman, & Cairney, 2014) first by calculating the interclass correlation (ICC) between the PPQ‐YP subscales completed at the two time points. The ICC scores ranged from .51 to .85, and this range of scores showed reasonable test–retest reliability. The second method was to test whether there was significant difference between the two time points and this was assessed through related‐samples Wilcoxon test. There were no significant differences between subscale total scores at each time point except for the scores on the symptoms associated with pain (pain identity subscale where *Z* = −2.79, *p *=* *.005; See Table 8). Spearman's Rho correlations between the PPQ‐YP at each time point was included and found that there was good stability over this period with most of the correlations being >.5 ranging from .37 to .71 (See Table 8). The values for the *recurrence* and the *predictability* subscales were <.5; however, there were no significant differences between time points for either of these subscales.

**Table 8:** Test–retest reliability over 2 weeks (Interclass correlations, Spearman's rho correlation, Wilcoxon test significance *n* = 43 to assess the test–retest reliability over 2‐week period)

PPQ‐YP subscale	ICC	Spearman's Rho correlation	Wilcoxon test significance
Consequences	.86	.71**	.70
Control	.74	.67**	.59
Coherence	.70	.55**	.60
Recurrence	.51	.30*	.24
Predictability	.58	.46**	.81
Responsive	.82	.69**	.77
Anticipatory	.68	.65**	.34
Symptoms (Identity)	.84	.65**	.06
Pain identity	.80	.60**	.01*
Treatment identity	.76	.64**	.41
Pain VAS	.79	.69**	.27

Note

*p < .05; **p <* *.001.

## Discussion

In their 2014 review, Law and colleagues raised the possibility that children and young people may be more likely to focus on the *symptoms* of their condition rather than on more sophisticated or complex representations of their long‐term conditions. In the current study, we found this to be the case for young individuals with a long‐term inflammatory condition. In response, we developed and completed the preliminary validation of an instrument to assess a range of pain beliefs in adolescents. The Pain Perceptions Questionnaire for Young People (PPQ‐YP) is the first tool to assess pain beliefs with adolescents with a chronic recurrent pain condition which corresponds to theoretically derived concepts which underpin the CS‐SRM framework (Leventhal *et al*., 1998). Its domains and items take account of social and developmental differences in the belief structures of adolescents from those of adults, an issue that has been raised by a range of theoreticians (Eccleston & Crombez, 2007; Gelman, 2009). Thus, while PPQ‐YP item domains correspond to most of those included in the Revised Illness Perceptions Questionnaire (IPQ‐R), the item content better reflects differences in the ways young people conceptualized their pain and have been written to ensure comprehension.

The final version contains 32 items assessing beliefs about consequences of pain, controllability, and understanding pain coherence and pain recurrence. Fourteen items assessed emotional representations, 15 items assessed identity and 17 items assessed the causal attributions. The PPQ‐YP underwent preliminary validation with a sample of adolescents with a long‐term condition, JIA.

The PPQ‐YP will facilitate the examination of a broader range of beliefs which could better predict adjustment to chronic pain. While the fear‐avoidance model highlighted the importance of cognitive appraisal, and may explain avoidance behaviour, it does not help to identify behaviours that could lead to adjustment.

Few other studies have examined how adolescents conceptualize their chronic pain condition. This research provides evidence to support the idea that children's and adolescents' conceptualization of a long‐term condition would differ from adult patients (Harbeck & Peterson, 1992; Huguet, Eccleston, Miro, & Gauntlett‐Gilbert, 2009). Unlike adults, adolescents in the current study were unlikely to consider emotions or psychological factors as having a causal role in pain. Conceptualizing psycho‐neuroimmunological processes is likely to involve more sophisticated personal pain models than those usually held by children and young people. This corresponds with findings from previous work where the belief least likely to be held by school children is that emotions may affect their pain experiences (Huguet *et al*., 2009). Despite this, the adolescents in the current study were able to recognize the duration and the emotional impact that pain had on them. For this reason, the PPQ‐YP included assessment of the frequency and intensity of emotional representations of their pain.

Importantly, the emotional representation subscales of the PPQ‐YP can differentiate between those adolescents who hold a responsive emotional representation and those with an anticipatory emotional representation. Previous work with pain diaries demonstrated that emotional variability predicts activity limitations which suggests that emotional representations could be important targets for interventions (Connelly *et al*., 2012).

The development of the PPQ‐YP is an important step in the systematic assessment of adolescents' conceptualization of their chronic pain experiences. It provides a means to track changes in both cognitive and emotional representations of chronic pain. This may mean that key targets for psychological interventions designed to improve pain outcomes could be identified. Further work may be required to validate the PPQ‐YP with other groups.

The CS‐SRM has provided an important framework for understanding and anticipating the cognitive drivers' of coping behaviours which occur in adults in response to the experience of a health threat (Leventhal *et al*., 2012). The current work has suggested important ways in which this model should be modified to incorporate differences in how younger people with a long‐term condition think about their illness experiences. Differences in adult and adolescent thinking have implications for researchers and clinicians in terms of assessing key illness beliefs, as well as for helping young people develop a coherent understanding of their condition, of treatment options or the lifestyle adjustments associated with best long‐term outcomes.

### Study strengths

The mixed methods used in this study enabled the researchers to interrogate existing constructs and to devise a questionnaire containing new items that are (1) relevant to adolescents, (2) appropriately reflect their developmental stage, and (3) assess their experiences of their condition. The use of mixed methods in the study, in particular the face‐to‐face interviews, meant the PPQ‐YP is not only underpinned by existing theory, but also informed by new insights and theoretical modifications derived from the qualitative work. Cognitive interviewing revealed the real‐time cognitive processes that participants utilized when answering items (Willis, 2004; Willis *et al*., 1991).

Furthermore, utilizing framework analysis allowed for both inductive and deductive explorations of concepts, something which benefits the process of theory development (Ritchie & Lewis, 2003) and resulted in the identification of important differences in the adolescents' constructs compared to those measured by the adult IPQ‐R. The data were organized and analysed according to an established theoretical framework, and this allowed for an exploration and identification of the predetermined mental representation constructs and their applicability to adolescents.

### Study limitations

A potential limitation of the study was that utilizing an established questionnaire could limit the exploration of the ways in which adolescents conceptualize pain. While it is not clear whether the items of the questionnaire limited the exploration, the adolescents were open about their reasoning in answering the items addressing both conceptual issues and methodological issues in measuring their beliefs.

In addition, cognitive interviewing depends on the abilities of the respondent to ‘think‐aloud’ which can feel artificial in nature (Drennan, 2003). However, the interviewer was highly experienced in undertaking interviews with young people, and well‐developed cognitive interviewing protocols were followed including verbal probing and think‐aloud methods. The extensive data set suggests that the respondents were able to participate fully.

The influence of parents remaining with their child during the interviews was considered while analysing the data. There were no suggestions from the data or the observation notes that the adolescents were monitoring their responses, but ultimately, the impact of the parent's presence is unknown. Furthermore, while we cannot be certain, we do not think that the study location (hospital clinic) led the participants to focus on pain. The study took place during routine clinical appointments rather than an appointment requested due to the exacerbation of symptoms.

The cause domain of the PPQ‐YP had the most missing data. We had included an option for participants to add their own cause to ensure that our final version includes relevant options; interestingly, only one of the adolescents wrote that they believed their recent pain was ‘caused by their arthritis’. The probable reason for this was that participants were asked for the cause of the most recent pain episode rather than the underlying cause of the pain. All those completing the PPQ‐YP had been diagnosed for at least a year, and it is possible that casual attributions change when trying to account for their most recent pain episode. However, the assessment of beliefs about the causes of recent pain flares is an area which needs to be examined further, and additional work into how this may change over time following initial diagnosis would be interesting. It will be useful to identify whether beliefs about causes of individual pain episodes better predict behaviour than beliefs about causes of the long‐term condition. Exploring this link has important implications for situations in which pain occurs in the absence of disease activity in conditions such as inflammatory arthritis, as well as in other pain conditions such as fibromyalgia.

The response rate in Phase 3 was 37%. This response rate may indicate a sample bias. However, one investigation of survey response rates for data collection of this sort described a postal response rate of this size as average (Baruch & Holtom, 2008). The PPQ‐YP was sent to potential participants with a large set of follow‐up questionnaires for another study (113 additional items) and the low response rate may simply reflect the participant burden involved in postal questionnaires. We would anticipate that the response rate to the PPQ‐YP would be higher when used on its own or alongside other short measures.

### Conclusions

This is the first study to evaluate the applicability of the CS‐SRM framework for use with adolescents with a long‐term condition and to use this to develop a new tool to assess the pain beliefs of adolescents with JIA, the PPQ‐YP. The use of real‐time cognitive interviewing facilitated the development of items that were relevant to the adolescents and reflective of their developmental stage and understanding of their condition. These modifications and developments were used to create a new tool, the PPQ‐YP which was validated with adolescents with JIA, and has implications for further research into pain beliefs and for clinical use.

## Conflict of interests

All authors declare no conflict of interest.

## Supporting information


**Table S1.** Frequency and problem category for each item of the IPQ‐R.

**Data S1.** Pain Perceptions Questionnaire.
