# Supplementary material for: The prioritization of symptom beliefs over illness beliefs: The development and validation of the Pain Perception Questionnaire for Young People
Source: Br J Health Psychol. 2017 Oct 9;23(1):68–87. doi: 10.1111/bjhp.12275 (PMC5765490; doi:10.1111/bjhp.12275)
Supplement: Supplementary file 1 — Table S1. Frequency and problem category for each item of the IPQ‐R. [file BJHP-23-68-s001.docx]

Supplementary Table 1: Frequency and problem category for each item of the IPQ-R

|  | IPQ-R Items | No problem | Not relevant | Incongruent endorsement | Incongruent  answer | Confusion | Total  problems |
| --- | --- | --- | --- | --- | --- | --- | --- |
| Timeline Chronicity | My arthritis will go away soon | 10 | 0 | 3 | 3 | 3 | 9 |
|  | Will be with my for the rest of my life | 12 | 0 | 6 | 0 | 0 | 6 |
|  | Will last a long time | 15 | 0 | 2 | 1 | 0 | 3 |
|  | Will soon get better | 9 | 0 | 4 | 4 | 1 | 9 |
|  | Will improve in time | 11 | 0 | 3 | 4 | 0 | 7 |
|  | Will last forever | 15 | 0 | 3 | 1 | 1 | 5 |
|  | TOTAL | 72 | 0 | 21 | 13 | 5 | **39** |
| Consequences | My arthritis is serious | 12 | 1 | 3 | 2 | 0 | 6 |
|  | Has a large effect on my life | 11 | 0 | 6 | 0 | 0 | 6 |
|  | Does not have much effect on my life | 17 | 1 | 0 | 1 | 0 | 2 |
|  | Affects what other people think of me | 8 | 2 | 1 | 3 | 4 | 10 |
|  | Costs a lot of money to my family or the hospitals | 7 | 6 | 2 | 1 | 3 | 12 |
|  | Makes difficulties for my family | 15 | 0 | 1 | 1 | 1 | 3 |
|  | TOTAL | 70 | 10 | 13 | 8 | 8 | **39** |
| Personal Control | There is a lot I can do to control my arthritis | 12 | 0 | 5 | 1 | 1 | 7 |
|  | What I do makes my arthritis better or worse | 7 | 0 | 4 | 3 | 4 | 11 |
|  | The way my arthritis acts depends on me | 8 | 0 | 5 | 2 | 3 | 10 |
|  | What I do now will not change how arthritis affects me in future | 4 | 1 | 2 | 6 | 5 | 14 |
|  | Nothing I do will have any effect on my arthritis | 9 | 0 | 7 | 0 | 2 | 9 |
|  | I have the power to have an effect on my arthritis | 7 | 1 | 4 | 4 | 3 | 12 |
|  | TOTAL | 44 | 2 | 27 | 16 | 18 | **63** |
| Treatment control | My treatment will cure my arthritis | 3 | 14 | 0 | 2 | 0 | 16 |
|  | There is very little that can be done to make my arthritis better | 16 | 0 | 0 | 1 | 1 | 2 |
|  | My treatment will prevent the bad effects of arthritis on me | 6 | 1 | 3 | 4 | 5 | 13 |
|  | Can help control my arthritis | 14 | 1 | 2 | 1 | 0 | 4 |
|  | Nothing can help my arthritis | 18 |  | 1 | 0 | 0 | 1 |
|  | TOTAL | 57 | 16 | 6 | 8 | 6 | **36** |
| Illness coherence | My symptoms are puzzling to me | 5 | 1 | 0 | 10 | 3 | 14 |
|  | My arthritis is a mystery to me | 14 | 2 | 0 | 1 | 1 | 4 |
|  | I understand my arthritis clearly | 12 | 0 | 5 | 1 |  | 6 |
|  | My arthritis doesn’t make any sense to me | 11 | 0 | 4 | 2 | 1 | 7 |
|  | I don’t understand my arthritis | 9 | 0 | 5 | 3 | 1 | 9 |
|  | TOTAL | 51 | 3 | 14 | 17 | 6 | **40** |
| Timeline cyclical | My arthritis changes everyday | 11 | 2 | 1 | 3 | 1 | 7 |
|  | My arthritis comes and goes in cycles | 9 | 6 | 0 | 2 | 4 | 12 |
|  | It is hard to tell what my arthritis will do next | 17 | 0 | 1 | 0 | 0 | 1 |
|  | My arthritis gets better and worse all the time | 10 | 2 | 1 | 5 | 0 | 7 |
|  | TOTAL | 47 | 10 | 2 | 10 | 5 | **27** |
| Emotional representation | I feel down and sad thinking about my arthritis | 15 | 2 | 1 | 0 | 0 | 3 |
|  | My arthritis makes me angry | 15 | 0 | 1 | 2 | 0 | 3 |
|  | My arthritis makes me upset | 12 | 1 | 3 | 2 | 0 | 6 |
|  | My arthritis makes me feel afraid | 16 | 0 | 1 | 1 | 0 | 2 |
|  | My arthritis does not worry me | 12 | 0 | 2 | 4 | 1 | 7 |
|  | My arthritis makes me nervous and anxious | 14 | 0 | 1 | 2 | 1 | 4 |
|  | TOTAL | 84 | 3 | 9 | 11 | 2 | **25** |
| Cause | Stress or worry | 3 | 7 | 0 | 4 | 5 | 16 |
|  | It runs in the family | 18 | 0 | 0 | 2 | 0 | 2 |
|  | A germ or virus | 12 | 2 | 2 | 3 | 1 | 8 |
|  | Diet or eating habits | 8 | 11 | 1 | 0 | 0 | 12 |
|  | Chance or bad luck | 7 | 2 | 8 | 3 | 0 | 13 |
|  | Poor health and bad medical care in my past | 13 | 4 | 0 | 1 | 2 | 7 |
|  | Pollution (like fumes, dirty water, and like) or toxins in the environment | 11 | 6 | 0 | 1 | 1 | 9 |
|  | My own behaviour | 11 | 6 | 1 | 0 | 2 | 9 |
|  | My attitude (for example thinking negatively) about life | 14 | 4 | 1 | 0 | 1 | 6 |
|  | Family problems, family worries | 11 | 8 | 0 | 0 | 1 | 9 |
|  | Doing too much | 12 | 1 | 2 | 5 | 0 | 8 |
|  | Feeling down, lonely, nervous or empty | 11 | 8 | 0 | 0 | 1 | 9 |
|  | Getting older | 7 | 10 | 0 | 3 | 0 | 13 |
|  | Drinking alcohol | 6 | 12 | 0 | 2 | 0 | 14 |
|  | Smoking | 7 | 12 | 0 | 1 | 0 | 13 |
|  | Accident or injury | 13 | 0 | 4 | 3 | 0 | 7 |
|  | Type of person that I am | 11 | 5 | 0 | 2 | 2 | 9 |
|  | Immune system in my body | 7 | 0 | 6 | 2 | 5 | 13 |
|  | TOTAL | 182 | 98 | 25 | 32 | 22 | **177** |
| Identity | Feeling Pain | 9 | 1 | 0 | 0 | 0 | 1 |
|  | Sore mouth/throat | 9 | 1 | 0 | 0 | 0 | 1 |
|  | Felt like vomiting | 8 | 1 | 1 | 0 | 0 | 2 |
|  | Couldn’t breathe well | 8 | 2 | 0 | 0 | 0 | 2 |
|  | Lost or put on weight | 7 | 1 | 2 | 0 | 0 | 3 |
|  | Feeling tired | 7 | 1 | 0 | 1 | 1 | 3 |
|  | My joints felt stiff | 8 | 1 | 0 | 1 | 0 | 2 |
|  | Sore eyes/not see well | 8 | 1 | 1 | 0 | 0 | 2 |
|  | Feeling unwell | 6 | 1 | 3 | 0 | 0 | 4 |
|  | Getting headaches | 10 | 0 | 0 | 0 | 0 | 0 |
|  | Could not sleep well | 9 | 1 | 0 | 0 | 0 | 1 |
|  | Upset tummy | 7 | 1 | 2 | 0 | 0 | 3 |
|  | Felt dizzy | 9 | 1 | 0 | 0 | 0 | 1 |
|  | Felt weak | 9 | 1 | 0 | 0 | 0 | 1 |
|  | TOTAL | 114 | 14 | 9 | 2 | 1 | 26 |
