# Supplementary material for: The prioritization of symptom beliefs over illness beliefs: The development and validation of the Pain Perception Questionnaire for Young People
Source: Br J Health Psychol. 2017 Oct 9;23(1):68–87. doi: 10.1111/bjhp.12275 (PMC5765490; doi:10.1111/bjhp.12275)
Supplement: Supplementary file 2 — Data S1. Pain Perceptions Questionnaire. [file BJHP-23-68-s002.docx]

Pain Perceptions Questionnaire

for Young People


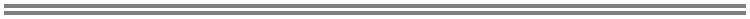


**PPQ-YP**

- We are interested in your views and how you feel about pain you may have relating to your [*condition – e.g. arthritis*]
- These are statements other people have made about their pain.
- For Section A, please show how much you agree or disagree with each of the following statements about your pain by marking it on the scale. Like this:

|  |  |  |  |  |  |  |  |  |
| --- | --- | --- | --- | --- | --- | --- | --- | --- |
|  |  |  |  |  |  |  |  |  |
|  |  |  |  |  |  |  |  |  |
|  | Disagree | | Neither agree  nor disagree | | Agree | |  |  |
|  |  |  |  |  |  |  |  |  |
|  |  |  |  |  |  |  |  |  |
| Strongly  disagree | | |  |  |  | Strongly  agree | | |


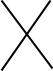


**Please tell us the date on which you completed this form**

**Date:**

1

**Section A**.

A1. I believe I will stop getting pain soon

|  |  |  |  |  |  |  |  |  |
| --- | --- | --- | --- | --- | --- | --- | --- | --- |
|  |  |  |  |  |  |  |  |  |
|  |  |  |  |  |  |  |  |  |
|  | Disagree | | Neither agree | | Agree | |  |  |
|  |  |  |  |  |  |  |  |  |
|  |  |  |  |  |  |  |  |  |
| Strongly | | | nor disagree | |  | Strongly | | |
| disagree | | |  |  |  | agree | | |
|  |  |  |  |  |  |  |  |  |

| A2. | I believe I will keep having pain when I am an adult |
| --- | --- |
|  |  |

|  |  |  |  |  |  |  |  |  |
| --- | --- | --- | --- | --- | --- | --- | --- | --- |
|  |  |  |  |  |  |  |  |  |
|  |  |  |  |  |  |  |  |  |
|  | Disagree | | Neither agree | | Agree | |  |  |
|  |  |  |  |  |  |  |  |  |
|  |  |  |  |  |  |  |  |  |
| Strongly | | | nor disagree | |  | Strongly | | |
| disagree | | |  |  |  | agree | | |
|  |  |  |  |  |  |  |  |  |

| A3. | When I get pain, it lasts a long time |
| --- | --- |
|  |  |

|  |  |  |  |  |  |  |  |  |
| --- | --- | --- | --- | --- | --- | --- | --- | --- |
|  |  |  |  |  |  |  |  |  |
|  |  |  |  |  |  |  |  |  |
|  | Disagree | | Neither agree | | Agree | |  |  |
|  |  |  |  |  |  |  |  |  |
|  |  |  |  |  |  |  |  |  |
| Strongly | | | nor disagree | |  | Strongly | | |
| disagree | | |  |  |  | agree | | |
|  |  |  |  |  |  |  |  |  |

| A4. | Over time I am getting pain more often |
| --- | --- |
|  |  |

|  |  |  |  |  |  |  |  |  |
| --- | --- | --- | --- | --- | --- | --- | --- | --- |
|  |  |  |  |  |  |  |  |  |
|  |  |  |  |  |  |  |  |  |
|  | Disagree | | Neither agree | | Agree | |  |  |
|  |  |  |  |  |  |  |  |  |
|  |  |  |  |  |  |  |  |  |
| Strongly | | | nor disagree | |  | Strongly | | |
| disagree | | |  |  |  | agree | | |
|  |  |  |  |  |  |  |  |  |

2

A5. When I get pain I think my pain will improve in

|  |  |  |  |  |  |  |  |  |  |
| --- | --- | --- | --- | --- | --- | --- | --- | --- | --- |
|  |  |  |  |  |  |  |  |  |  |
|  |  |  |  |  |  |  |  |  |  |
|  |  | A day or | | A week or less | | Two weeks or | |  |  |
| An hour or | |  |  |  |  |  |  | More | |
|  |  | less | |  |  |  | less |  |  |
| less | |  |  |  |  |  |  | than two | |
|  |  |  |  |  |  |  |  |  |  |
|  |  |  |  |  |  |  |  | weeks | |

A6. When I get pain it makes me think my pain is

|  |  |  |  |  |  |  |  |  |  |
| --- | --- | --- | --- | --- | --- | --- | --- | --- | --- |
|  |  |  |  |  |  |  |  |  |  |
|  |  |  |  |  |  |  |  |  |  |
|  |  | A bit | | serious | | Somewhat | |  |  |
| Not serious | |  |  |  |  |  |  | Very | |
|  |  | serious | |  |  |  | serious |  |  |
| at all | |  |  |  |  |  |  | serious | |
|  |  |  |  |  |  |  |  |  |  |

A7. When I have pain it stops me from taking part in activities such as PE

|  |  |  |  |  |  |  |  |  |
| --- | --- | --- | --- | --- | --- | --- | --- | --- |
|  |  |  |  |  |  |  |  |  |
|  |  |  |  |  |  |  |  |  |
|  | Disagree | | Neither agree | | Agree | |  |  |
|  |  |  |  |  |  |  |  |  |
|  |  |  |  |  |  |  |  |  |
| Strongly | | | nor disagree | |  | Strongly | | |
| disagree | | |  |  |  | agree | | |
|  |  |  |  |  |  |  |  |  |

A8. When I have pain, it affects me at school such as school work, school friends

|  |  |  |  |  |  |  |  |  |
| --- | --- | --- | --- | --- | --- | --- | --- | --- |
|  |  |  |  |  |  |  |  |  |
|  |  |  |  |  |  |  |  |  |
|  | Disagree | | Neither agree | | Agree | |  |  |
|  |  |  |  |  |  |  |  |  |
|  |  |  |  |  |  |  |  |  |
| Strongly | | | nor disagree | |  | Strongly | | |
| disagree | | |  |  |  | agree | | |
|  |  |  |  |  |  |  |  |  |

3

A9. When I have pain, it affects how I am at home

|  |  |  |  |  |  |  |  |  |
| --- | --- | --- | --- | --- | --- | --- | --- | --- |
|  |  |  |  |  |  |  |  |  |
|  |  |  |  |  |  |  |  |  |
|  | Disagree | | Neither agree | | Agree | |  |  |
|  |  |  |  |  |  |  |  |  |
|  |  |  |  |  |  |  |  |  |
| Strongly | | | nor disagree | |  | Strongly | | |
| disagree | | |  |  |  | agree | | |
|  |  |  |  |  |  |  |  |  |

A10. I believe my pain affects what other people think of me

|  |  |  |  |  |  |  |  |  |
| --- | --- | --- | --- | --- | --- | --- | --- | --- |
|  |  |  |  |  |  |  |  |  |
|  |  |  |  |  |  |  |  |  |
|  | Disagree | | Neither agree | | Agree | |  |  |
|  |  |  |  |  |  |  |  |  |
|  |  |  |  |  |  |  |  |  |
| Strongly | | | nor disagree | |  | Strongly | | |
| disagree | | |  |  |  | agree | | |
|  |  |  |  |  |  |  |  |  |

A11. I believe my family spend more money because I have pain

|  |  |  |  |  |  |  |  |  |
| --- | --- | --- | --- | --- | --- | --- | --- | --- |
|  |  |  |  |  |  |  |  |  |
|  |  |  |  |  |  |  |  |  |
|  | Disagree | | Neither agree | | Agree | |  |  |
|  |  |  |  |  |  |  |  |  |
|  |  |  |  |  |  |  |  |  |
| Strongly | | | nor disagree | |  | Strongly | | |
| disagree | | |  |  |  | agree | | |
|  |  |  |  |  |  |  |  |  |

A12. I believe my pain makes hospitals spend a lot of money

|  |  |  |  |  |  |  |  |  |
| --- | --- | --- | --- | --- | --- | --- | --- | --- |
|  |  |  |  |  |  |  |  |  |
|  |  |  |  |  |  |  |  |  |
|  | Disagree | | Neither agree | | Agree | |  |  |
|  |  |  |  |  |  |  |  |  |
|  |  |  |  |  |  |  |  |  |
| Strongly | | | nor disagree | |  | Strongly | | |
| disagree | | |  |  |  | agree | | |
|  |  |  |  |  |  |  |  |  |

4

A13. I believe my pain causes problems for my family

|  |  |  |  |  |  |  |  |  |
| --- | --- | --- | --- | --- | --- | --- | --- | --- |
|  |  |  |  |  |  |  |  |  |
|  |  |  |  |  |  |  |  |  |
|  | Disagree | | Neither agree | | Agree | |  |  |
|  |  |  |  |  |  |  |  |  |
|  |  |  |  |  |  |  |  |  |
| Strongly | | | nor disagree | |  | Strongly | | |
| disagree | | |  |  |  | agree | | |
|  |  |  |  |  |  |  |  |  |

| A14. | I can do a lot to control my pain |
| --- | --- |
|  |  |

|  |  |  |  |  |  |  |  |  |
| --- | --- | --- | --- | --- | --- | --- | --- | --- |
|  |  |  |  |  |  |  |  |  |
|  |  |  |  |  |  |  |  |  |
|  | Disagree | | Neither agree | | Agree | |  |  |
|  |  |  |  |  |  |  |  |  |
|  |  |  |  |  |  |  |  |  |
| Strongly | | | nor disagree | |  | Strongly | | |
| disagree | | |  |  |  | agree | | |
|  |  |  |  |  |  |  |  |  |

A15. This is the amount of control I feel I have over my pain

|  |  |  |  |  |  |  |  |  |  |
| --- | --- | --- | --- | --- | --- | --- | --- | --- | --- |
|  |  |  |  |  |  |  |  |  |  |
|  |  |  |  |  |  |  |  |  |  |
|  |  | Some | | Quite a bit of | | A lot of | |  |  |
| No control at | |  |  |  |  |  |  | Full | |
|  |  | control | | control | | control | |  |  |
| all | |  |  |  |  |  |  | control | |
|  |  |  |  |  |  |  |  |  |  |

A16. There are things I can do to make my pain better

|  |  |  |  |  |  |  |  |  |
| --- | --- | --- | --- | --- | --- | --- | --- | --- |
|  |  |  |  |  |  |  |  |  |
|  |  |  |  |  |  |  |  |  |
|  | Disagree | | Neither agree | | Agree | |  |  |
|  |  |  |  |  |  |  |  |  |
|  |  |  |  |  |  |  |  |  |
| Strongly | | | nor disagree | |  | Strongly | | |
| disagree | | |  |  |  | agree | | |
|  |  |  |  |  |  |  |  |  |

5

A17. Things I do now can affect whether I have pain in the future

|  |  |  |  |  |  |  |  |  |
| --- | --- | --- | --- | --- | --- | --- | --- | --- |
|  |  |  |  |  |  |  |  |  |
|  |  |  |  |  |  |  |  |  |
|  | Disagree | | Neither agree | | Agree | |  |  |
|  |  |  |  |  |  |  |  |  |
|  |  |  |  |  |  |  |  |  |
| Strongly | | | nor disagree | |  | Strongly | | |
| disagree | | |  |  |  | agree | | |
|  |  |  |  |  |  |  |  |  |

A18. Taking my treatment means I have

|  |  |  |  |  |  |  |  |  |  |
| --- | --- | --- | --- | --- | --- | --- | --- | --- | --- |
|  |  |  |  |  |  |  |  |  |  |
|  |  |  |  |  |  |  |  |  |  |
|  |  | Some  control over  my pain | | Quite a bit of  control over my  pain | | A lot of control  over my pain | |  |  |
| No control over  my pain | |  |  |  |  |  |  | Full control  over my pain | |
|  |  |  |  |  |  |  |  |  |  |

A19. I feel confused about why I get pain

|  |  |  |  |  |  |  |  |  |
| --- | --- | --- | --- | --- | --- | --- | --- | --- |
|  |  |  |  |  |  |  |  |  |
|  |  |  |  |  |  |  |  |  |
|  | Disagree | | Neither agree | | Agree | |  |  |
|  |  |  |  |  |  |  |  |  |
|  |  |  |  |  |  |  |  |  |
| Strongly | | | nor disagree | |  | Strongly | | |
| disagree | | |  |  |  | agree | | |
|  |  |  |  |  |  |  |  |  |

A20. I understand how my treatment for pain works

|  |  |  |  |  |  |  |  |  |
| --- | --- | --- | --- | --- | --- | --- | --- | --- |
|  |  |  |  |  |  |  |  |  |
|  |  |  |  |  |  |  |  |  |
|  | Disagree | | Neither agree | | Agree | |  |  |
|  |  |  |  |  |  |  |  |  |
|  |  |  |  |  |  |  |  |  |
| Strongly | | | nor disagree | |  | Strongly | | |
| disagree | | |  |  |  | agree | | |
|  |  |  |  |  |  |  |  |  |

A21. I understand my pain clearly

|  |  |  |  |  |  |  |  |  |
| --- | --- | --- | --- | --- | --- | --- | --- | --- |
|  |  |  |  |  |  |  |  |  |
|  |  |  |  |  |  |  |  |  |
|  | Disagree | | Neither agree | | Agree | |  |  |
|  |  |  |  |  |  |  |  |  |
|  |  |  |  |  |  |  |  |  |
| Strongly | | | nor disagree | |  | Strongly | | |
| disagree | | |  |  |  | agree | | |
|  |  |  |  |  |  |  |  |  |

A22. I don’t have any questions about my pain

|  |  |  |  |  |  |  |  |  |
| --- | --- | --- | --- | --- | --- | --- | --- | --- |
|  |  |  |  |  |  |  |  |  |
|  |  |  |  |  |  |  |  |  |
|  | Disagree | | Neither agree | | Agree | |  |  |
|  |  |  |  |  |  |  |  |  |
|  |  |  |  |  |  |  |  |  |
| Strongly | | | nor disagree | |  | Strongly | | |
| disagree | | |  |  |  | agree | | |
|  |  |  |  |  |  |  |  |  |

A23. When I have pain I understand what causes my pain

|  |  |  |  |  |  |  |  |  |
| --- | --- | --- | --- | --- | --- | --- | --- | --- |
|  |  |  |  |  |  |  |  |  |
|  |  |  |  |  |  |  |  |  |
|  | Disagree | | Neither agree | | Agree | |  |  |
|  |  |  |  |  |  |  |  |  |
|  |  |  |  |  |  |  |  |  |
| Strongly | | | nor disagree | |  | Strongly | | |
| disagree | | |  |  |  | agree | | |
|  |  |  |  |  |  |  |  |  |

A24. My pain changes everyday

|  |  |  |  |  |  |  |  |  |
| --- | --- | --- | --- | --- | --- | --- | --- | --- |
|  |  |  |  |  |  |  |  |  |
|  |  |  |  |  |  |  |  |  |
|  | Disagree | | Neither agree | | Agree | |  |  |
|  |  |  |  |  |  |  |  |  |
|  |  |  |  |  |  |  |  |  |
| Strongly | | | nor disagree | |  | Strongly | | |
| disagree | | |  |  |  | agree | | |
|  |  |  |  |  |  |  |  |  |

7

| A25. | My pain comes and goes |
| --- | --- |
|  |  |

|  |  |  |  |  |  |  |  |  |
| --- | --- | --- | --- | --- | --- | --- | --- | --- |
|  |  |  |  |  |  |  |  |  |
|  |  |  |  |  |  |  |  |  |
|  | Disagree | | Neither agree | | Agree | |  |  |
|  |  |  |  |  |  |  |  |  |
|  |  |  |  |  |  |  |  |  |
| Strongly | | | nor disagree | |  | Strongly | | |
| disagree | | |  |  |  | agree | | |
|  |  |  |  |  |  |  |  |  |

| A26. | I can predict when I will get pain |
| --- | --- |
|  |  |

|  |  |  |  |  |  |  |  |  |
| --- | --- | --- | --- | --- | --- | --- | --- | --- |
|  |  |  |  |  |  |  |  |  |
|  |  |  |  |  |  |  |  |  |
|  | Disagree | | Neither agree | | Agree | |  |  |
|  |  |  |  |  |  |  |  |  |
|  |  |  |  |  |  |  |  |  |
| Strongly | | | nor disagree | |  | Strongly | | |
| disagree | | |  |  |  | agree | | |
|  |  |  |  |  |  |  |  |  |

A27. I can see a pattern in how and when I get pain

|  |  |  |  |  |  |  |  |  |
| --- | --- | --- | --- | --- | --- | --- | --- | --- |
|  |  |  |  |  |  |  |  |  |
|  |  |  |  |  |  |  |  |  |
|  | Disagree | | Neither agree | | Agree | |  |  |
|  |  |  |  |  |  |  |  |  |
|  |  |  |  |  |  |  |  |  |
| Strongly | | | nor disagree | |  | Strongly | | |
| disagree | | |  |  |  | agree | | |
|  |  |  |  |  |  |  |  |  |

A28. I am in control of my treatment for my pain

|  |  |  |  |  |  |  |  |  |
| --- | --- | --- | --- | --- | --- | --- | --- | --- |
|  |  |  |  |  |  |  |  |  |
|  |  |  |  |  |  |  |  |  |
|  | Disagree | | Neither agree | | Agree | |  |  |
|  |  |  |  |  |  |  |  |  |
|  |  |  |  |  |  |  |  |  |
| Strongly | | | nor disagree | |  | Strongly | | |
| disagree | | |  |  |  | agree | | |
|  |  |  |  |  |  |  |  |  |

8

A29. My treatment helps my pain get better

|  |  |  |  |  |  |  |  |  |
| --- | --- | --- | --- | --- | --- | --- | --- | --- |
|  |  |  |  |  |  |  |  |  |
|  |  |  |  |  |  |  |  |  |
|  | Disagree | | Neither agree | | Agree | |  |  |
|  |  |  |  |  |  |  |  |  |
|  |  |  |  |  |  |  |  |  |
| Strongly | | | nor disagree | |  | Strongly | | |
| disagree | | |  |  |  | agree | | |
|  |  |  |  |  |  |  |  |  |

| A30. | My treatment protects me from pain |
| --- | --- |
|  |  |

|  |  |  |  |  |  |  |  |  |
| --- | --- | --- | --- | --- | --- | --- | --- | --- |
|  |  |  |  |  |  |  |  |  |
|  |  |  |  |  |  |  |  |  |
|  | Disagree | | Neither agree | | Agree | |  |  |
|  |  |  |  |  |  |  |  |  |
|  |  |  |  |  |  |  |  |  |
| Strongly | | | nor disagree | |  | Strongly | | |
| disagree | | |  |  |  | agree | | |
|  |  |  |  |  |  |  |  |  |

| A31. | I can continue with my activities because of my treatment |
| --- | --- |
|  |  |

|  |  |  |  |  |  |  |  |  |
| --- | --- | --- | --- | --- | --- | --- | --- | --- |
|  |  |  |  |  |  |  |  |  |
|  |  |  |  |  |  |  |  |  |
|  | Disagree | | Neither agree | | Agree | |  |  |
|  |  |  |  |  |  |  |  |  |
|  |  |  |  |  |  |  |  |  |
| Strongly | | | nor disagree | |  | Strongly | | |
| disagree | | |  |  |  | agree | | |
|  |  |  |  |  |  |  |  |  |

9

**Section B.**

We would like to ask you about how you FEEL when you are in pain Show how often you feel this way by marking on the scale

|  |  |  |  |  |  |  |  |  |
| --- | --- | --- | --- | --- | --- | --- | --- | --- |
|  |  |  |  |  |  |  |  |  |
|  |  |  |  |  |  |  |  |  |
|  | Rarely | | Sometimes | | Most of | |  |  |
|  |  |  |  |  |  |  | Always | |
|  |  |  |  |  |  |  |  |  |
| Never | | |  |  | the time | |  |  |
|  |  |  |  |  |  |  |  |  |


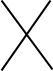


And when do you feel these emotions how much do you feel them

|  |  |  |  |  |  |  |  |  |
| --- | --- | --- | --- | --- | --- | --- | --- | --- |
|  |  |  |  |  |  |  |  |  |
|  |  |  |  |  |  |  |  |  |
|  | A little | | Quite a bit | | A fair | | A lot |  |
|  |  |  |  |  |  |  |  |  |
|  |  |  |  |  |  |  |  |  |
| None | | |  |  | amount | |  |  |
|  |  |  |  |  |  |  |  |  |


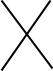


B1. When I am in pain I feel down and sad

How often?

| Rarely | Sometimes | Most of | Always |
| --- | --- | --- | --- |
| Never |  | the time |  |
|  |  |  |  |

How much?

| A little | Quite a bit | A fair | A lot |
| --- | --- | --- | --- |
| None |  | amount |  |
|  |  |  |  |

B2. When I am in pain I feel angry

How often?

| Rarely | Sometimes | Most of | Always |
| --- | --- | --- | --- |
| Never |  | the time |  |
|  |  |  |  |

How much?

| A little | Quite a bit | A fair | A lot |
| --- | --- | --- | --- |
| None |  | amount |  |
|  |  |  |  |

B3. When I am in pain I feel upset

How often?

| Rarely | Sometimes | Most of | Always |
| --- | --- | --- | --- |
| Never |  | the time |  |
|  |  |  |  |

How much?

| A little | Quite a bit | A fair | A lot |
| --- | --- | --- | --- |
| None |  | amount |  |
|  |  |  |  |

B4. When I am in pain I feel afraid

How often?

| Rarely | Sometimes | Most of | Always |
| --- | --- | --- | --- |
| Never |  | the time |  |
|  |  |  |  |

How much?

| A little | Quite a bit | A fair | A lot |
| --- | --- | --- | --- |
| None |  | amount |  |
|  |  |  |  |

B5. When I am in pain I feel frustrated

How often?

| Rarely | Sometimes | Most of | Always |
| --- | --- | --- | --- |
| Never |  | the time |  |
|  |  |  |  |

How much?

| A little | Quite a bit | A fair | A lot |
| --- | --- | --- | --- |
| None |  | amount |  |
|  |  |  |  |

B6. When I am in pain I feel worried

How often?

| Rarely | Sometimes | Most of | Always |
| --- | --- | --- | --- |
| Never |  | the time |  |
|  |  |  |  |

How much?

| A little | Quite a bit | A fair | A lot |
| --- | --- | --- | --- |
| None |  | amount |  |
|  |  |  |  |

B7. When I am in pain I feel anxious

How often?

| Rarely | Sometimes | Most of | Always |
| --- | --- | --- | --- |
| Never |  | the time |  |
|  |  |  |  |

How much?

|  |  |  |  |  |  |  |  |  |
| --- | --- | --- | --- | --- | --- | --- | --- | --- |
|  |  |  |  |  |  |  |  |  |
|  |  |  |  |  |  |  |  |  |
|  | A little | | Quite a bit | | A fair | | A lot |  |
|  |  |  |  |  |  |  |  |  |
|  |  |  |  |  |  |  |  |  |
| None | | |  |  | amount | |  |  |
|  |  |  |  |  |  |  |  |  |

11

**Section C.**

We would like to ask you about any SYMPTOMS you may have experienced since you have been having pain.

- Please show whether you have experienced each of the following symptoms in the PAST WEEK by circling YES
- For each please show whether you believe it is related to your pain by circling YES and whether you believe it is related to your treatment by circling YES
- If you have not felt the symptoms please circle NO and move on to the next symptom

|  | **Symptom** | **I had this recently** | | **If answer is YES** | **This symptom is related** | | **This symptom is related to** | |
| --- | --- | --- | --- | --- | --- | --- | --- | --- |
|  |  |  |  |  | **to my Pain** |  | **my treatment** |  |
| C1 | Felt like vomiting | NO | YES | IF YES | YES | NO | YES | NO |
|  |  |  |  |  |  |  |  |  |
| C2 | Couldn’t breathe well | NO | YES | IF YES | YES | NO | YES | NO |
|  |  |  |  |  |  |  |  |  |
| C3 | Lost or put on weight | NO | YES | IF YES | YES | NO | YES | NO |
|  |  |  |  |  |  |  |  |  |
| C4 | Feeling tired | NO | YES | IF YES | YES | NO | YES | NO |
|  |  |  |  |  |  |  |  |  |
| C5 | Joints feel stiff | NO | YES | IF YES | YES | NO | YES | NO |
|  |  |  |  |  |  |  |  |  |
| C6 | Joints feel sore | NO | YES | IF YES | YES | NO | YES | NO |
|  |  |  |  |  |  |  |  |  |
| C7 | Sore eyes | NO | YES | IF YES | YES | NO | YES | NO |
|  | /not see well |  |  |  |  |  |  |  |
|  |  |  |  |  |  |  |  |  |
| C8 | Feeling unwell | NO | YES | IF YES | YES | NO | YES | NO |
|  |  |  |  |  |  |  |  |  |
| C9 | Getting headaches | NO | YES | IF YES | YES | NO | YES | NO |
|  |  |  |  |  |  |  |  |  |
| C10 | Could not sleep well | NO | YES | IF YES | YES | NO | YES | NO |
|  |  |  |  |  |  |  |  |  |
| C11 | Upset tummy | NO | YES | IF YES | YES | NO | YES | NO |
|  |  |  |  |  |  |  |  |  |
| C12 | Felt dizzy | NO | YES | IF YES | YES | NO | YES | NO |
|  |  |  |  |  |  |  |  |  |
| C13 | Felt weak | NO | YES | IF YES | YES | NO | YES | NO |
|  |  |  |  |  |  |  |  |  |
| C14 | Feelings of tightness in | NO | YES | IF YES | YES | NO | YES | NO |
|  | my body |  |  |  |  |  |  |  |
|  |  |  |  |  |  |  |  |  |
| C15 | Change in my mood | NO | YES | IF YES | YES | NO | YES | NO |
|  |  |  |  |  |  |  |  |  |


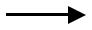

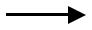

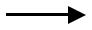

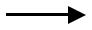

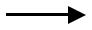

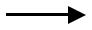

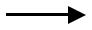

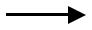

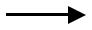

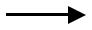

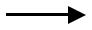

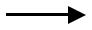

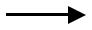

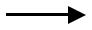

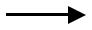


12

**Section D.**

We are interested in your views about what causes your pain. The list below gives examples suggested by other young people.

| • | FIRST Circle the THREE causes you believe caused your most recent pain | | |
| --- | --- | --- | --- |
|  | *For example*: | stress or worry |  |
| • | THEN Place a number near the circled causes in the order of importance with | | |
|  | **1** meaning the most important. | |  |
| *For example*: If you think that runs in the family is the most important cause of | | | |
|  | your pain, then you would number this 1. Like this: | | |
|  |  | stress or worry | 3. |
|  |  | runs in the family | 1. |
|  |  | immune system | 2. |
|  |  |  |  |
| D1. | Stress or worry |  |  |
| D2. | It runs in the family (genetics) | |  |
| D3. | A germ or virus |  |  |
| D4. | Diet or eating habits |  |  |
| D5. | Chance or bad luck |  |  |
| D6. | Poor health and bad medical care in my past | | |
| D7. | Pollution (like fumes, dirty water) or toxins in the environment | | |
| D8. | My own behaviour |  |  |
| D9. | My attitude (for example thinking negatively) about life | | |
| D10. | Family problems, family worries | |  |
| D11. | Doing too much |  |  |
| D12. | Feeling down, lonely, nervous or empty | |  |
| D13. | Getting older |  |  |
| D14. | Accident or injury |  |  |
| D15. | The type of person that I am | |  |
| D16. | My immune system |  |  |
| D17 | *Any other cause that you think of* | |  |
|  |  |  |  |


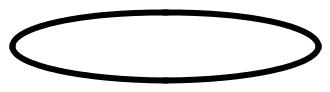

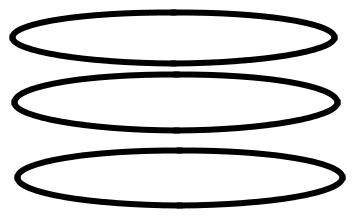


In the PAST WEEK how much pain have you had because of your [*arthritis*]? Place a mark on the line below, to indicate the severity of the pain

| 0 | 100 |
| --- | --- |
| No pain | Very severe pain |

14
